# (*R*)-*N*-(Biphenyl-4-yl)-*tert*-butane­sulfinamide

**DOI:** 10.1107/S1600536812015127

**Published:** 2012-04-13

**Authors:** Binbin Zhang, Yan Wang, Xiaofei Sun, Wenguo Wang, Qingle Zeng

**Affiliations:** a State Key Lab of Geohazard Prevention and Geoenvironment Protection and Institute of Green Catalysis and Synthesis, College of Materials and Chemistry and Chemical Engineering, Chengdu University of Technology, Chengdu 610059, People’s Republic of China; b Fujian Institute of Research on the Structure of Matter, Chinese Academy of Sciences, Fuzhou 350002, People’s Republic of China

## Abstract

In the title compound, C_16_H_19_NOS, the dihedral angle between the two aromatic rings is 38.98 (8)°. The crystal structure is stabilized by N—H⋯O hydrogen bonds, which link neighbouring mol­ecules into chains running parallel to the *a* axis.

## Related literature

For related structures, see: Sun *et al.* (2012 ▶); Jasinski *et al.* (2012 ▶); Gainsford *et al.* (2011 ▶).
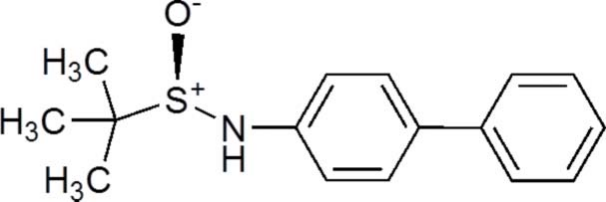


## Experimental

### 

#### Crystal data

C_16_H_19_NOS
*M* *_r_* = 273.38
Orthorhombic, 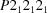

*a* = 9.3588 (5) Å
*b* = 11.9452 (5) Å
*c* = 13.3136 (7) Å
*V* = 1488.36 (12) Å^3^
*Z* = 4
Mo *K*α radiation
μ = 0.21 mm^−1^
*T* = 293 K
0.43 × 0.41 × 0.40 mm

#### Data collection

Oxford Diffraction Xcalibur Eos diffractometer
Absorption correction: multi-scan (*CrysAlis PRO*; Oxford Diffraction, 2010 ▶) *T*_min_ = 0.988, *T*_max_ = 1.000
3983 measured reflections
2766 independent reflections
2325 reflections with *I* > 2σ(*I*)
*R*_int_ = 0.019

#### Refinement

*R*[*F* ^2^ > 2σ(*F* ^2^)] = 0.047
*wR*(*F* ^2^) = 0.097
*S* = 1.06
2766 reflections
175 parameters
H-atom parameters constrained
Δρ_max_ = 0.19 e Å^−3^
Δρ_min_ = −0.22 e Å^−3^
Absolute structure: assigned from the known absolute structure of the (*R*)-tert-butanesulfinamide starting material; the Flack (1983 ▶) parameter is consistent with this assignment, 1017 Friedel pairs
Flack parameter: 0.03 (10)

### 

Data collection: *CrysAlis PRO* (Oxford Diffraction, 2010 ▶); cell refinement: *CrysAlis PRO*; data reduction: *CrysAlis PRO*; program(s) used to solve structure: *SHELXS97* (Sheldrick, 2008 ▶); program(s) used to refine structure: *SHELXL97* (Sheldrick, 2008 ▶); molecular graphics: *OLEX2* (Dolomanov *et al.*, 2009 ▶); software used to prepare material for publication: *OLEX2*.

## Supplementary Material

Crystal structure: contains datablock(s) I, global. DOI: 10.1107/S1600536812015127/rz2735sup1.cif

Structure factors: contains datablock(s) I. DOI: 10.1107/S1600536812015127/rz2735Isup2.hkl

Supplementary material file. DOI: 10.1107/S1600536812015127/rz2735Isup3.cdx

Supplementary material file. DOI: 10.1107/S1600536812015127/rz2735Isup4.cml

Additional supplementary materials:  crystallographic information; 3D view; checkCIF report

## Figures and Tables

**Table 1:** Hydrogen-bond geometry (Å, °)

*D*—H⋯*A*	*D*—H	H⋯*A*	*D*⋯*A*	*D*—H⋯*A*
N1—H1⋯O1^i^	0.86	2.35	3.144 (3)	154

Symmetry code: (i) 
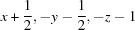
.

**Fig. 1.**
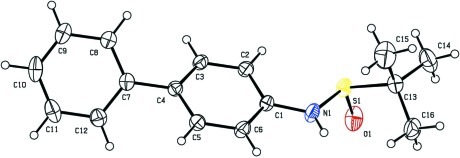
Molecular structure of (I), with atom labels and 50% probability displacement ellipsoids for non-H atoms.

**Fig. 2.**
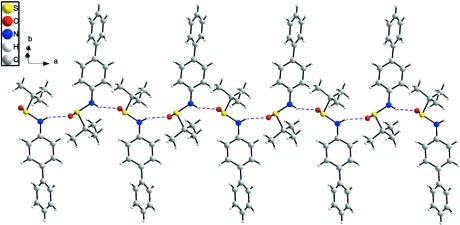
The one-dimensional structure of (I) in the crystal packing, showing intermolecular hydrogen bonding as dashed lines.

## supplementary crystallographic information

### Comment

Sulfinamides, especially chiral sulfinamides, are an important class of organic compounds in modern organic chemistry, and a great number of such compounds have been synthesized. In our continuous study on chiral *N*-aryl-*tert*-butanesulfinamides (Sun *et al.*, 2012), we have prepared the title compound and report its crystal structure herein.

In the molecule of the title compound (Fig. 1) the aromatic rings of the biphenyl are tilted to form a dihedral angle of 38.98 (8)°, which is comparable to the value observed in other related compounds containing the biphenyl group (Jasinski *et al.*, 2012; Gainsford *et al.*, 2011). In the crystal packing (Fig. 2), the molecules are linked by intermolecular N—H···O hydrogen bonds (Table 1) into one-dimensional chains running parallel to the the *a* axis.

### Experimental

A oven-dried ground test tube, which was equipped with a magnetic stir bar and fitted with a rubber septum, was charged with (*R*)-*tert*-butanesulfinamide (0.121 g, 1.0 mmol), Pd2(dba)3 (0.018 g, 0.02 mmol; dba is dibenzylideneacetone), 2-di-*tert*-butylphosphino-2',4',6'-triisopropylbiphenyl (0.0212 g, 0.05 mmol) and NaOH (0.08 g, 2 mmol). The vessel was evacuated and backfilled with argon three times, then 4-biphenyl bromide (1.3 mmol), toluene (10 ml) and degassed water (0.3 ml) were added *via* syringe. The solution was stirred at 90°C for 20 h. The reaction mixture was then cooled to room temperature, quenched by water, and extracted with chloroform (20 ml) for twice. The organic layers were combined, and dried over anhydrous sodium sulfate and filtrated. The filterate was condensed under vacuum. The residual was purified with silica gel column chromatography with a solution of petroleum ether and ethyl acetate (5:1 *v*/*v*) as eluent to give the title compound (*R*)-*N*-(4-biphenyl)-*tert*-butanesulfinamide. A test tube containing a petroleum ether and ethyl acetate (1:1 *v*/*v*) solution of the title compound was covered with a piece of filter paper and placed motionless at room temperature, and a single-crystal was cultured in the bottom of the test tube. Spectroscopic analysis: ^1^H NMR (300 MHz, CDCl_3_), δ (ppm): 7.49–7.29 (m, 7H), 7.06 (d, J = 8.5 Hz, 2H), 6.03 (d, J = 3.9 Hz, 1H), 1.37 (s, 9H). ^13^C NMR (300 MHz, CDCl_3_), δ (ppm): 114.6, 140.4, 135.6, 128.6, 127.9, 126.8, 126.6, 118.4, 56.5, 22.4. FT—IR (KBr) (cm^-1^): 3453, 3252, 2926, 1610, 1519, 1485, 1386, 1305, 1286, 1268, 1228, 1191, 1057, 912, 880, 838, 767. [α]D = -110.8 (c 0.15, ethyl acetate). ESI-MS (negative mode), m/*z* = 272 [M—H]-. Anal. Calcd for C_16_H_19_NOS: C, 70.29; H, 7.00; N, 5.12. Found: C, 70.43; H, 7.16; N 5.01.

### Refinement

All H atoms were positioned geometrically and refined using a riding model, with N—H = 0.86 Å, C—H = 0.93–0.96 Å, and with *U*_iso_(H) = 1.2 *U*_eq_(C, N) or 1.5 *U*_eq_(C) for methyl H atoms.

### Crystal data

**Table:**

C_16_H_19_NOS	*D*_x_ = 1.220 Mg m^−^^3^
*M**_r_* = 273.38	Melting point: 427 K
Orthorhombic, *P*2_1_2_1_2_1_	Mo *K*α radiation, λ = 0.7107 Å
Hall symbol: P 2ac 2ab	Cell parameters from 1425 reflections
*a* = 9.3588 (5) Å	θ = 3.1–28.9°
*b* = 11.9452 (5) Å	µ = 0.21 mm^−^^1^
*c* = 13.3136 (7) Å	*T* = 293 K
*V* = 1488.36 (12) Å^3^	Block, colourless
*Z* = 4	0.43 × 0.41 × 0.40 mm
*F*(000) = 584	

### Data collection

**Table:**

Oxford Diffraction Xcalibur Eos diffractometer	2766 independent reflections
Radiation source: Enhance (Mo) X-ray Source	2325 reflections with *I* > 2σ(*I*)
Graphite monochromator	*R*_int_ = 0.019
Detector resolution: 16.0874 pixels mm^-1^	θ_max_ = 26.4°, θ_min_ = 3.1°
ω scans	*h* = −11→5
Absorption correction: multi-scan (*CrysAlis PRO*; Oxford Diffraction, 2010)	*k* = −7→14
*T*_min_ = 0.988, *T*_max_ = 1.000	*l* = −16→16
3983 measured reflections	

### Refinement

**Table:**

Refinement on *F*^2^	Secondary atom site location: difference Fourier map
Least-squares matrix: full	Hydrogen site location: inferred from neighbouring sites
*R*[*F*^2^ > 2σ(*F*^2^)] = 0.047	H-atom parameters constrained
*wR*(*F*^2^) = 0.097	*w* = 1/[σ^2^(*F*_o_^2^) + (0.0358*P*)^2^ + 0.0448*P*] where *P* = (*F*_o_^2^ + 2*F*_c_^2^)/3
*S* = 1.06	(Δ/σ)_max_ < 0.001
2766 reflections	Δρ_max_ = 0.19 e Å^−^^3^
175 parameters	Δρ_min_ = −0.22 e Å^−^^3^
0 restraints	Absolute structure: assigned from the known absolute structure of the (*R*)-tert-butanesulfinamide starting material; the Flack (1983) parameter is consistent with this assignment, 1017 Friedel pairs
Primary atom site location: structure-invariant direct methods	Flack parameter: 0.03 (10)

### Special details

**Table:**

Geometry. All e.s.d.'s (except the e.s.d. in the dihedral angle between two l.s. planes) are estimated using the full covariance matrix. The cell e.s.d.'s are taken into account individually in the estimation of e.s.d.'s in distances, angles and torsion angles; correlations between e.s.d.'s in cell parameters are only used when they are defined by crystal symmetry. An approximate (isotropic) treatment of cell e.s.d.'s is used for estimating e.s.d.'s involving l.s. planes.
Refinement. Refinement of *F*^2^ against ALL reflections. The weighted *R*-factor *wR* and goodness of fit *S* are based on *F*^2^, conventional *R*-factors *R* are based on *F*, with *F* set to zero for negative *F*^2^. The threshold expression of *F*^2^ > σ(*F*^2^) is used only for calculating *R*-factors(gt) *etc*. and is not relevant to the choice of reflections for refinement. *R*-factors based on *F*^2^ are statistically about twice as large as those based on *F*, and *R*- factors based on ALL data will be even larger.

### Fractional atomic coordinates and isotropic or equivalent isotropic displacement parameters (Å^2^)

**Table:**

	*x*	*y*	*z*	*U*_iso_*/*U*_eq_	
S1	−0.15256 (8)	−0.31264 (5)	−0.40407 (5)	0.04256 (19)	
O1	−0.2124 (3)	−0.22979 (16)	−0.47627 (16)	0.0639 (7)	
N1	−0.0065 (3)	−0.36901 (17)	−0.45167 (18)	0.0488 (7)	
H1	0.0702	−0.3294	−0.4536	0.059*	
C1	−0.0021 (3)	−0.4790 (2)	−0.48927 (19)	0.0355 (6)	
C2	−0.1153 (3)	−0.5532 (2)	−0.4810 (2)	0.0415 (7)	
H2	−0.1987	−0.5318	−0.4482	0.050*	
C3	−0.1032 (3)	−0.6600 (2)	−0.5220 (2)	0.0400 (7)	
H3	−0.1801	−0.7090	−0.5166	0.048*	
C4	0.0197 (3)	−0.6959 (2)	−0.57082 (17)	0.0357 (6)	
C5	0.1315 (3)	−0.6196 (2)	−0.5777 (2)	0.0395 (6)	
H5	0.2149	−0.6404	−0.6107	0.047*	
C6	0.1217 (3)	−0.5131 (2)	−0.5366 (2)	0.0423 (7)	
H6	0.1990	−0.4644	−0.5410	0.051*	
C7	0.0310 (3)	−0.8089 (2)	−0.61585 (18)	0.0377 (6)	
C8	−0.0253 (3)	−0.9024 (2)	−0.5693 (2)	0.0476 (8)	
H8	−0.0694	−0.8944	−0.5071	0.057*	
C9	−0.0181 (4)	−1.0075 (2)	−0.6126 (2)	0.0558 (8)	
H9	−0.0581	−1.0688	−0.5801	0.067*	
C10	0.0480 (4)	−1.0208 (3)	−0.7031 (3)	0.0600 (9)	
H10	0.0535	−1.0914	−0.7324	0.072*	
C11	0.1060 (3)	−0.9301 (3)	−0.7507 (2)	0.0586 (9)	
H11	0.1514	−0.9395	−0.8122	0.070*	
C12	0.0980 (3)	−0.8240 (2)	−0.7081 (2)	0.0471 (7)	
H12	0.1375	−0.7630	−0.7414	0.057*	
C13	−0.0712 (3)	−0.2310 (2)	−0.3024 (2)	0.0461 (7)	
C14	−0.1958 (4)	−0.1746 (3)	−0.2498 (2)	0.0769 (12)	
H14A	−0.2385	−0.1207	−0.2941	0.115*	
H14B	−0.1622	−0.1375	−0.1903	0.115*	
H14C	−0.2656	−0.2299	−0.2316	0.115*	
C15	−0.0002 (6)	−0.3136 (3)	−0.2314 (3)	0.0911 (14)	
H15A	−0.0670	−0.3713	−0.2138	0.137*	
H15B	0.0301	−0.2752	−0.1718	0.137*	
H15C	0.0812	−0.3465	−0.2639	0.137*	
C16	0.0316 (4)	−0.1448 (2)	−0.3427 (3)	0.0670 (10)	
H16A	0.1088	−0.1818	−0.3767	0.100*	
H16B	0.0690	−0.1013	−0.2880	0.100*	
H16C	−0.0173	−0.0965	−0.3889	0.100*	

### Atomic displacement parameters (Å^2^)

**Table:**

	*U*^11^	*U*^22^	*U*^33^	*U*^12^	*U*^13^	*U*^23^
S1	0.0340 (4)	0.0355 (3)	0.0581 (4)	0.0019 (3)	−0.0013 (4)	−0.0073 (4)
O1	0.0696 (17)	0.0521 (12)	0.0701 (14)	0.0165 (12)	−0.0276 (13)	−0.0088 (11)
N1	0.0381 (15)	0.0330 (11)	0.0754 (16)	−0.0062 (11)	0.0102 (13)	−0.0137 (11)
C1	0.0374 (16)	0.0289 (13)	0.0403 (14)	0.0022 (12)	−0.0009 (13)	−0.0009 (11)
C2	0.0343 (16)	0.0364 (14)	0.0538 (16)	0.0000 (13)	0.0130 (14)	−0.0031 (13)
C3	0.0377 (16)	0.0319 (14)	0.0505 (16)	−0.0043 (12)	0.0086 (14)	−0.0013 (12)
C4	0.0386 (15)	0.0326 (12)	0.0360 (13)	0.0026 (13)	−0.0008 (12)	0.0012 (12)
C5	0.0306 (15)	0.0389 (13)	0.0488 (16)	0.0043 (12)	0.0058 (14)	−0.0017 (13)
C6	0.0363 (17)	0.0353 (14)	0.0552 (17)	−0.0038 (13)	0.0004 (14)	−0.0009 (13)
C7	0.0333 (14)	0.0373 (13)	0.0423 (14)	0.0061 (13)	−0.0057 (12)	−0.0024 (13)
C8	0.0546 (19)	0.0393 (14)	0.0488 (17)	−0.0007 (15)	0.0038 (16)	−0.0023 (13)
C9	0.063 (2)	0.0360 (14)	0.068 (2)	−0.0014 (16)	−0.0094 (19)	−0.0060 (15)
C10	0.054 (2)	0.0503 (18)	0.075 (2)	0.0092 (17)	−0.021 (2)	−0.0296 (18)
C11	0.046 (2)	0.073 (2)	0.0567 (19)	0.0086 (19)	−0.0018 (16)	−0.0280 (18)
C12	0.0446 (18)	0.0491 (16)	0.0477 (16)	0.0017 (15)	−0.0003 (15)	−0.0044 (15)
C13	0.0484 (19)	0.0452 (15)	0.0447 (16)	0.0038 (14)	−0.0030 (15)	−0.0074 (14)
C14	0.075 (3)	0.085 (3)	0.070 (2)	0.006 (2)	0.014 (2)	−0.030 (2)
C15	0.125 (4)	0.076 (2)	0.073 (2)	0.018 (3)	−0.036 (3)	−0.002 (2)
C16	0.065 (2)	0.0593 (19)	0.077 (2)	−0.0194 (18)	0.001 (2)	−0.0224 (18)

### Geometric parameters (Å, º)

**Table:**

S1—O1	1.489 (2)	C9—H9	0.9300
S1—N1	1.650 (2)	C9—C10	1.364 (4)
S1—C13	1.833 (3)	C10—H10	0.9300
N1—H1	0.8600	C10—C11	1.367 (4)
N1—C1	1.407 (3)	C11—H11	0.9300
C1—C2	1.385 (3)	C11—C12	1.391 (4)
C1—C6	1.381 (4)	C12—H12	0.9300
C2—H2	0.9300	C13—C14	1.519 (4)
C2—C3	1.393 (3)	C13—C15	1.519 (4)
C3—H3	0.9300	C13—C16	1.508 (4)
C3—C4	1.389 (4)	C14—H14A	0.9600
C4—C5	1.390 (4)	C14—H14B	0.9600
C4—C7	1.481 (3)	C14—H14C	0.9600
C5—H5	0.9300	C15—H15A	0.9600
C5—C6	1.388 (3)	C15—H15B	0.9600
C6—H6	0.9300	C15—H15C	0.9600
C7—C8	1.381 (4)	C16—H16A	0.9600
C7—C12	1.390 (4)	C16—H16B	0.9600
C8—H8	0.9300	C16—H16C	0.9600
C8—C9	1.383 (4)		
			
O1—S1—N1	109.58 (13)	C9—C10—H10	120.1
O1—S1—C13	106.21 (12)	C9—C10—C11	119.8 (3)
N1—S1—C13	99.00 (13)	C11—C10—H10	120.1
S1—N1—H1	118.6	C10—C11—H11	119.6
C1—N1—S1	122.8 (2)	C10—C11—C12	120.8 (3)
C1—N1—H1	118.6	C12—C11—H11	119.6
C2—C1—N1	123.1 (3)	C7—C12—C11	120.2 (3)
C6—C1—N1	117.5 (2)	C7—C12—H12	119.9
C6—C1—C2	119.3 (2)	C11—C12—H12	119.9
C1—C2—H2	120.2	C14—C13—S1	105.0 (2)
C1—C2—C3	119.5 (3)	C14—C13—C15	109.7 (3)
C3—C2—H2	120.2	C15—C13—S1	107.2 (2)
C2—C3—H3	118.9	C16—C13—S1	111.5 (2)
C4—C3—C2	122.2 (3)	C16—C13—C14	110.6 (3)
C4—C3—H3	118.9	C16—C13—C15	112.6 (3)
C3—C4—C5	116.8 (2)	C13—C14—H14A	109.5
C3—C4—C7	122.0 (2)	C13—C14—H14B	109.5
C5—C4—C7	121.2 (2)	C13—C14—H14C	109.5
C4—C5—H5	119.1	H14A—C14—H14B	109.5
C6—C5—C4	121.7 (3)	H14A—C14—H14C	109.5
C6—C5—H5	119.1	H14B—C14—H14C	109.5
C1—C6—C5	120.4 (3)	C13—C15—H15A	109.5
C1—C6—H6	119.8	C13—C15—H15B	109.5
C5—C6—H6	119.8	C13—C15—H15C	109.5
C8—C7—C4	121.9 (2)	H15A—C15—H15B	109.5
C8—C7—C12	117.6 (2)	H15A—C15—H15C	109.5
C12—C7—C4	120.5 (2)	H15B—C15—H15C	109.5
C7—C8—H8	119.1	C13—C16—H16A	109.5
C7—C8—C9	121.9 (3)	C13—C16—H16B	109.5
C9—C8—H8	119.1	C13—C16—H16C	109.5
C8—C9—H9	120.1	H16A—C16—H16B	109.5
C10—C9—C8	119.8 (3)	H16A—C16—H16C	109.5
C10—C9—H9	120.1	H16B—C16—H16C	109.5

### Hydrogen-bond geometry (Å, º)

**Table:**

*D*—H···*A*	*D*—H	H···*A*	*D*···*A*	*D*—H···*A*
N1—H1···O1^i^	0.86	2.35	3.144 (3)	154

Symmetry code: (i) *x*+1/2, −*y*−1/2, −*z*−1.
